# High resolution structural and functional MRI of the hippocampus in young adults with Down syndrome

**DOI:** 10.1093/braincomms/fcab088

**Published:** 2021-04-19

**Authors:** Katherine A Koenig, Se-Hong Oh, Melissa R Stasko, Elizabeth C Roth, H Gerry Taylor, Stephen Ruedrich, Z Irene Wang, James B Leverenz, Alberto C S Costa

**Affiliations:** 1 Imaging Sciences, Imaging Institute, Cleveland Clinic, Cleveland, OH 44195, USA; 2 Department of Biomedical Engineering, Hankuk University of Foreign Studies, Yongin 449-791, Republic of Korea; 3 Department of Pediatrics, Case Western Reserve University, Cleveland, OH 44106, USA; 4 Abigail Wexner Research Institute at Nationwide Children’s Hospital, and Department of Pediatrics, The Ohio State University, Columbus, OH 43215, USA; 5 Department of Psychiatry, University Hospitals, Cleveland, OH 44106, USA; 6 Epilepsy Center, Neurological Institute, Cleveland Clinic, Cleveland, OH 44195, USA; 7 Lou Ruvo Center for Brain Health, Neurological Institute, Cleveland Clinic, Cleveland, OH 44195, USA

**Keywords:** Down syndrome, functional connectivity, hippocampus, MRI, spatial memory

## Abstract

Down syndrome is the phenotypic consequence of trisomy 21, with clinical presentation including both neurodevelopmental and neurodegenerative components. Although the intellectual disability typically displayed by individuals with Down syndrome is generally global, it also involves disproportionate deficits in hippocampally-mediated cognitive processes. Hippocampal dysfunction may also relate to Alzheimer’s disease-type pathology, which can appear in as early as the first decade of life and becomes universal by age 40. Using 7-tesla MRI of the brain, we present an assessment of the structure and function of the hippocampus in 34 individuals with Down syndrome (mean age 24.5 years ± 6.5) and 27 age- and sex-matched typically developing healthy controls. In addition to increased whole-brain mean cortical thickness and lateral ventricle volumes (*P *<* *1.0 × 10^−4^), individuals with Down syndrome showed selective volume reductions in bilateral hippocampal subfields cornu Ammonis field 1, dentate gyrus, and tail (*P *<* *0.005). In the group with Down syndrome, bilateral hippocampi showed widespread reductions in the strength of functional connectivity, predominately to frontal regions (*P *<* *0.02). Age was not related to hippocampal volumes or functional connectivity measures in either group, but both groups showed similar relationships of age to whole-brain volume measures (*P *<* *0.05). Finally, we performed an exploratory analysis of a subgroup of individuals with Down syndrome with both imaging and neuropsychological assessments. This analysis indicated that measures of spatial memory were related to mean cortical thickness, total grey matter volume and right hemisphere hippocampal subfield volumes (*P* < 0.02). This work provides a first demonstration of the usefulness of high-field MRI to detect subtle differences in structure and function of the hippocampus in individuals with Down syndrome, and suggests the potential for development of MRI-derived measures as surrogate markers of drug efficacy in pharmacological studies designed to investigate enhancement of cognitive function.

## Teaser

Individuals with Down syndrome show distinct deficits in cognitive processes that are mediated by the hippocampus. Using high-resolution MRI, Koenig *et al*. find volume reductions of select hippocampal subfields and widespread changes in synchronicity of the hippocampus in individuals with Down syndrome.

## Graphical Abstract

**Graphical Abstract:**
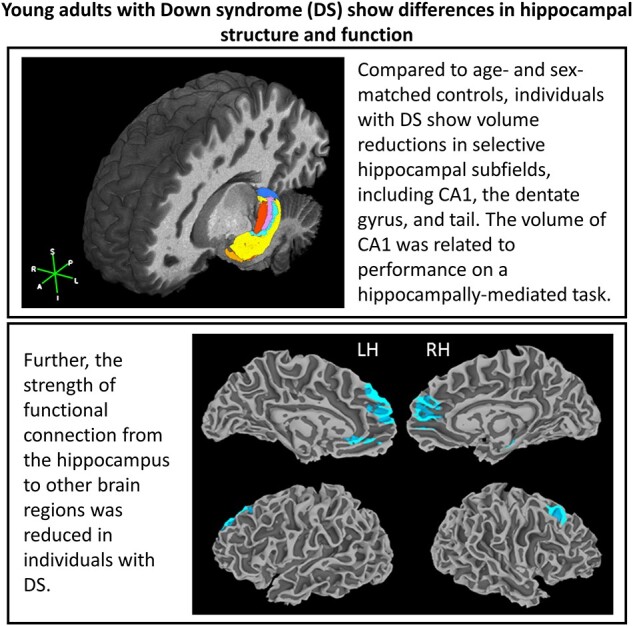

## Introduction

Down syndrome (DS) is the most prevalent of the genetic disorders that give rise to cognitive impairment, with 95% of cases caused by trisomy of chromosome 21 (T21). Individuals with DS show well-described physical characteristics and are at an increased risk for a long list of comorbidities, such as thyroid dysfunction, congenital heart disease and Alzheimer’s disease-like dementia.^1–3^ Cognitive function is variable in those with DS, but moderate intellectual disability is common.^4^ Although DS-associated cognitive impairment is generally global in nature, disproportionate deficits in expressive language,^5^^,^^6^ verbal short-term memory^7^ and hippocampally-mediated long-term memory^8^^,^^9^ have been described. Neuroanatomical characteristics of DS include decreased brain volume, delayed myelination, decreased dendritic arborization and regional reductions in the size of the cerebellum, prefrontal cortex and hippocampus.^10–14^

The hippocampal formation is an intricate, elongated structure that runs along the anterior–posterior axis of the medial temporal lobe. It is composed of architectonically distinct subregions that correspond to the flow of hippocampal input and output. It is involved in multiple cognitive functions including episodic memory^15^ and spatial processing.^16^ In addition to reductions in overall hippocampal size,^11^^,^^12^ histological studies provide evidence that, in DS, different hippocampal subfields may be differently impacted. Compared to controls, brains of foetuses with T21 show increased phagocytic activity in cornu Ammonis field 1 (CA1) and subiculum (SUB),^17^ and decreased thickness, reduced percentage of neurons, and reduced cell density in the SUB.^18^ An analysis of the hippocampal formation (including CA1 and the SUB) and the dentate gyrus (DG) in foetal brains with T21 showed lower volume and cell number in both regions compared to controls, but similar cell density.^19^ In addition, the DG shows reduced cell proliferation^20^ and decreased density of myelinated axons in the hilar region.^21^

Hippocampal atrophy is a common finding in non-DS Alzheimer’s disease, and dementia-related hippocampal atrophy has also been reported in DS.^22^^,^^23^ In non-DS Alzheimer’s disease and mild cognitive impairment (MCI), hippocampal subfields CA1 and the SUB tend to show the first signs of neuropathology, including neuronal loss and the development of neurofibrillary tangles.^24^ In cognitively preserved elderly without DS, CA1 and the SUB showed volume loss in those who later converted to MCI,^25^ and a sample of cognitively preserved elderly with amyloid-β pathology showed smaller volumes of the SUB, presubiculum, and hippocampal tail.^26^ These findings suggest that early development of Alzheimer’s disease neuropathology leads to preferential volume loss in specific hippocampal subfields, and also suggest another potential mechanism for hippocampal dysfunction in DS. Increased levels of amyloid precursor protein have been reported in foetal T21 hippocampi,^27^ with amyloid plaques found in the CA1 region even in the first decade of life.^28^ Widespread distribution of amyloid plaques in the hippocampus has been shown to occur after age 30, with the development of hippocampal neurofibrillary tangles after age 40.^29^

In addition to structural changes, multiple studies report altered function of the hippocampal formation in non-DS Alzheimer’s disease and MCI.^30–32^ The hippocampus has strong structural and functional connections to the posterior cingulate cortex (PCC), which are reported to decrease in strength in Alzheimer’s disease and MCI.^30^^,^^33^^,^^34^ The PCC is a key component of the default mode network (DMN), itself widely reported to be disrupted in clinical and preclinical Alzheimer’s disease.^35^^,^^36^ Although we found no reports describing hippocampal functional connectivity in individuals with DS, a decrease in global connectivity of the PCC was reported.^37^ Recent work in the Ts65Dn mouse model of DS found that young mice showed increased synchronization between the hippocampus and prefrontal regions at rest, and disruptions in hippocampal–prefrontal interaction during memory acquisition and retrieval and object familiarization.^38^

In this work, we use high resolution MRI to measure hippocampal subfield volumes and functional connectivity in teenagers and young adults with DS and in a sample of age-matched typical controls. We hypothesize that, compared to controls, individuals with DS will show reductions in the size of CA1, the SUB and the DG. We also investigate functional connectivity of the hippocampus, hypothesizing reduced strength of connection to the PCC in those with DS.

## Materials and methods

### Participants

Participants included 37 teenagers and non-demented adults with DS, determined by a medical diagnosis of T21 [mean age (years) 24.0 ± 6.6, range 15–35; 23 males] and 27 age-matched healthy controls (mean age 24.9 ± 6.1, range 15–36; 17 males). Prior to data collection, participants were consented in accordance with the Declaration of Helsinki. Data included in this analysis were collected under three Cleveland Clinic Institutional Review Board-approved protocols (141523; 16390; 13058). Detailed information regarding the composition of participants from each protocol can be found in the Supplementary materials. The three protocols ran concurrently, and all participants were scanned on the same MRI scanner using identical sequences. For all three protocols, MRI data collection occurred in a single scanning session.

Prior to enrolment, all participants (and their caregivers, if applicable) were interviewed to assess eligibility for study participation. Exclusion criteria for all participants included: (i) History of major psychiatric disorder such as schizophrenia, bipolar disorder, autism, Alzheimer’s disease or major depressive disorder; (ii) History of neurologic diagnosis such as traumatic brain injury, stroke or a diagnosis of seizure disorder in the past three years; (iii) Cognitive or physical limitations that resulted in the inability to complete study procedures; (iv) Confirmed clinical symptoms of dementia; and (v) MRI-specific exclusion criteria.

### Behavioural testing

All participants with DS met with a psychiatrist specializing in developmental disabilities for clinical assessment, which included medical history, a psychiatric diagnostic interview with the participant and caregiver, and mental status examination. Particular attention was paid to changes that might be related to dementia. No participants were found to have symptoms of dementia.

Individuals with DS who participated under Protocol 2 underwent neuropsychological testing. The test battery used here was previously described,^39^ and is detailed in the Supplementary materials. These measures were used in a supplementary analysis of the relationship of cognition to anatomical volumes.

### MR imaging

All participants were scanned on a Siemens 7T Magnetom scanner with SC72 gradient (Siemens Medical Solutions, Erlangen) using a head-only CP transmit and 32-channel phased-array receive coil (Nova Medical, Wilmington). Respiratory and cardiac fluctuations were measured using a plethysmograph and respiratory bellows during scanning. A whole-brain, T_1_-weighted MP2RAGE (0.75 mm^3^ isotropic voxel size) was acquired for all participants. A sub-sample of participants underwent a resting state functional connectivity scan. (132 repetitions, 81 axial slices, voxel size 0.75 × 0.75 × 1.5 mm^3^, TE/TR = 21 ms/2800 ms, matrix 160 × 160, FOV 210 × 210 mm^2^, receive bandwidth = 1562 Hz/pixel, scan time 7 min). Directly prior to the resting state scan, the scanner technician talked to the participants, making sure they were awake and asking them to keep their eyes closed for the next scan.

### Volumetric analysis

**Figure 1:**
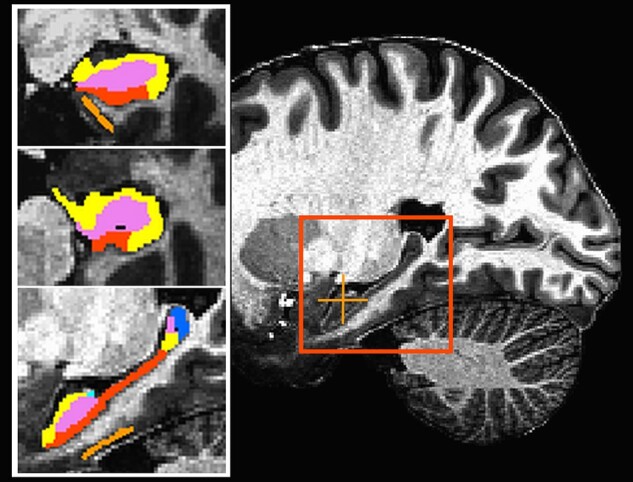
**Representative slices from the image used to calculate anatomical volumes**. The inset at left shows the same image with the hippocampal segmentation overlaid. The orange crosshair marks the location of the coronal (top) and axial (middle) slices. The red square outlines the enlarged sagittal slice at bottom. orange = entorhinal cortex; red = subiculum; yellow = CA1; light blue = CA2/CA3; violet = dentate gyrus; blue = tail.

To assess whole-brain volumes, mean volume of the left and right lateral ventricles, mean cortical thickness, and volumes of cerebral white matter (WM) and total and subcortical grey matter (GM) were calculated using the MP2RAGE processed using Freesurfer 6.0.^40^ Using the MP2RAGE, hippocampal subfield volumes were calculated using the Automated Segmentation of Hippocampal Subfields (ASHS) software^41^ and the ASHS 1.0 Compatible 7T atlas.^42^ ASHS returns intracranial volume (ICV) and volumes for the entorhinal cortex, SUB, CA1, CA2, CA3, DG and tail (Fig. 1). To account for differences in ICV, and for the sake of consistency, ASHS ICV measures were used to correct both ASHS and Freesurfer volumes. Corrected volumes were calculated as: (volume/ICV)*100.

### Statistical analysis

Unpaired *t*-tests compared ICV-corrected volumes between groups, and linear correlations were used to test the relationship of age to volume in each group. The false discovery rate (FDR) was used to adjust for multiple comparisons.^43^

Within-group differences in hemispheric asymmetry were determined by entering left and right ICV-corrected volumes into a paired Student’s *t*-test. Between-group differences in hemispheric asymmetry were assessed using the asymmetry index (AI). Using uncorrected hippocampal volumes, AI was calculated for each measure as: [(left—right)/(left + right)]*100. Unpaired *t*-tests compared AI between groups, with FDR adjustment.

### Functional connectivity

#### Post-processing

The first 4 volumes of the functional time series were removed. RETROICOR was used to regress out measured cardiac and respiratory signals at the voxel level.^44^ Concurrently, volumetric and slice-wise motion was regressed using SLOMOCO.^45^ The data were spatially filtered using a 2 mm filter,^46^ detrended, and fluctuations above 0.08 Hz were removed. The MP2RAGE was aligned to the functional volume using the AFNI program align_epi_anat.py.^47^ A supplementary analysis, undertaken to assess the impact of individual motion estimates on our results, is described in the Supplementary materials.

#### Seed selection

**Figure 2:**
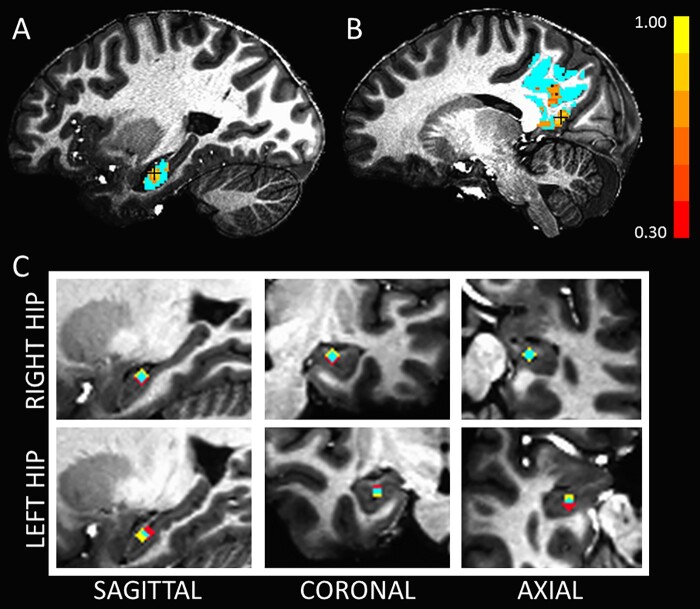
**Hippocampal seed definition in a representative subject**. (**A** and **B**) Functional connectivity from the head of the hippocampus within the specified ROI (shown in cyan). For display purposes, the correlation map is thresholded at Pearson’s correlation coefficient >0.30.(**A**) Functional connectivity in the head of the hippocampus, with the black crosshair noting the location of the voxel with the highest cross-correlation to the PCC. (**B**) Functional connectivity of the PCC ROI measured from the seed location in **A**. The black crosshair notes the location of the voxel with the highest cross-correlation to the hippocampus. Panel **C** shows the average location of the hippocampal ROIs for the control (red; *n* = 22) and DS (yellow; *n* = 22) groups. The overlap is shown in cyan. HIP, hippocampus.

Although our functional data are high resolution, the undulating and sometimes thin hippocampal structure contributes to a risk of inclusion of signals measured from non-brain tissue. To mitigate this risk, we chose to undertake a seed-based functional analysis focussed on the head of the hippocampus. This region is relatively thick, and includes large portions of the subfields of interest (CA1, the SUB and the DG). Using both anatomical and functional data, right and left seed locations in the head of the hippocampus were identified in native space for each participant. First, the head of the hippocampus was identified anatomically on the MP2RAGE using a previously described method.^48^ Next, the Yeo 7 network functional connectivity cortical parcellation,^49^ available in Freesurfer, was used to define the DMN. In this parcellation, Network 7 is identified as most representative of the DMN. Bilateral regions of interest (ROIs) from Network 7, covering the PCC and precuneus regions, were combined with individual-subject cortical ROIs from Freesurfer to create a conjunction mask representing GM voxels within the PCC. The hippocampal ROIs and PCC mask were aligned to the functional volume and used to create a masked functional time series containing only the hippocampal and PCC regions. For each hemisphere, the masked functional time series was used to identify the final hippocampal seed. The time series of each voxel in the head of the hippocampus was cross-correlated to that of each voxel in the conjunction mask, to identify the hippocampal voxel with the highest correlation to GM of the PCC (Fig. 2). That hippocampal voxel was taken as the center of a 9-voxel in-plane ROI, which represented the seed used in the connectivity analysis.

#### Connectivity map creation

For each participant, whole-brain functional connectivity maps were created for the left and right hippocampus. The mean time series of each seed was linearly detrended and correlated with the linearly detrended time series of each voxel located within brain tissue. Each correlation was converted to a Student’s *t*-score and the whole-brain distribution was normalized to unit variance and zero mean in order to correct for individual variations in global signal.^50^ The resulting connectivity maps represent the strength of left and right hippocampi to the whole brain.

**Figure 3:**
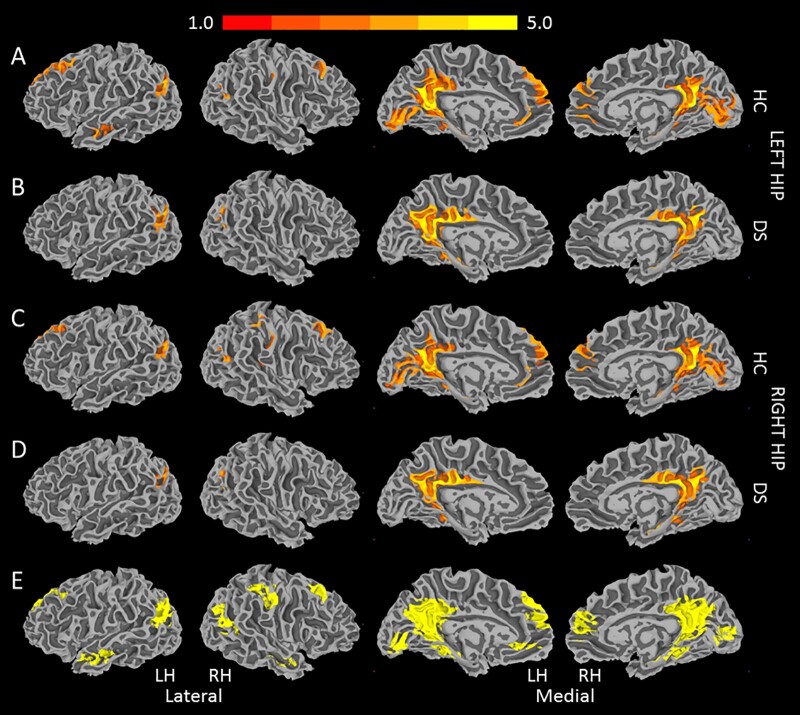
**Average functional connectivity of the hippocampus**. Average functional connectivity *z*-maps for: (**A**) left hippocampus, controls; (**B**) left hippocampus, DS; (**C**) right hippocampus, controls; (**D**) right hippocampus, DS. Single voxel threshold *P* < 0.0005, cluster size 500 (*n* = 22 in each group). (**E**) Conjunction mask used to define ROIs for group analysis. DS, Down syndrome; HC, healthy control; HIP, hippocampus; LH, left hemisphere; RH, right hemisphere.

Individuals with DS are known to have specific neuroanatomical differences compared to controls, which may introduce bias to analyses in common space. For this reason, we chose to measure our final connectivity values in native space using an ROI mask rather than undertake a whole-brain voxel-wise analysis in common space. By necessity, creation of the ROI mask first required the transformation of individual connectivity maps to Talairach space, which were then averaged by group. Figure 3A–D shows each of the four group-averaged maps: left and right hippocampus in DS and controls (single voxel threshold *P *<* *0.0005, cluster size 500). The left and right hippocampal maps showed similar connectivity patterns in each group, so the four maps in Fig. 3 were added to create a conjunction mask of regions that were significantly connected to the right or left hippocampus in either group (Fig. 3E). For each participant, the conjunction mask was transformed to individual space and the mean *z*-score was calculated for each ROI in the mask.

#### Statistical analysis

Unpaired *t*-tests were used to compare connectivity between groups. Linear correlations were used to test the relationship of age to connectivity measures in each group. In regions that showed group differences in connectivity strength, linear correlation was used to assess the relationship of functional connectivity to hippocampal volumes. The FDR was used to adjust for multiple comparisons in each of the analyses above.

### Data availability

The data that support the findings of this study are available on request from the corresponding author, dependent on a formal data sharing agreement with the administering institution.

## Results

### Volumetric analysis

#### Sample

The final sample for the brain volume analysis was 34 individuals with DS [mean age (years) 24.5 ± 6.5, range 15–35; 22 males] and 27 controls (described above). There were no group differences in age (*P *=* *0.817) or sex (*P *=* *0.888). Due to motion during scanning, three DS participants from Protocol 2 did not have MP2RAGE scans of sufficient quality to run using the ASHS software, and were excluded from further analysis (see Supplementary material for details).

Supplementary Table 1 shows cognitive scores for participants with DS in Protocol 2. An exploratory analysis of the relationship between volumetric and cognitive measures was undertaken for the 18 individuals with both data types, detailed in Supplementary materials: cognitive data analysis and in Supplementary Tables 1 and 2.

#### Group differences

**Figure 4:**
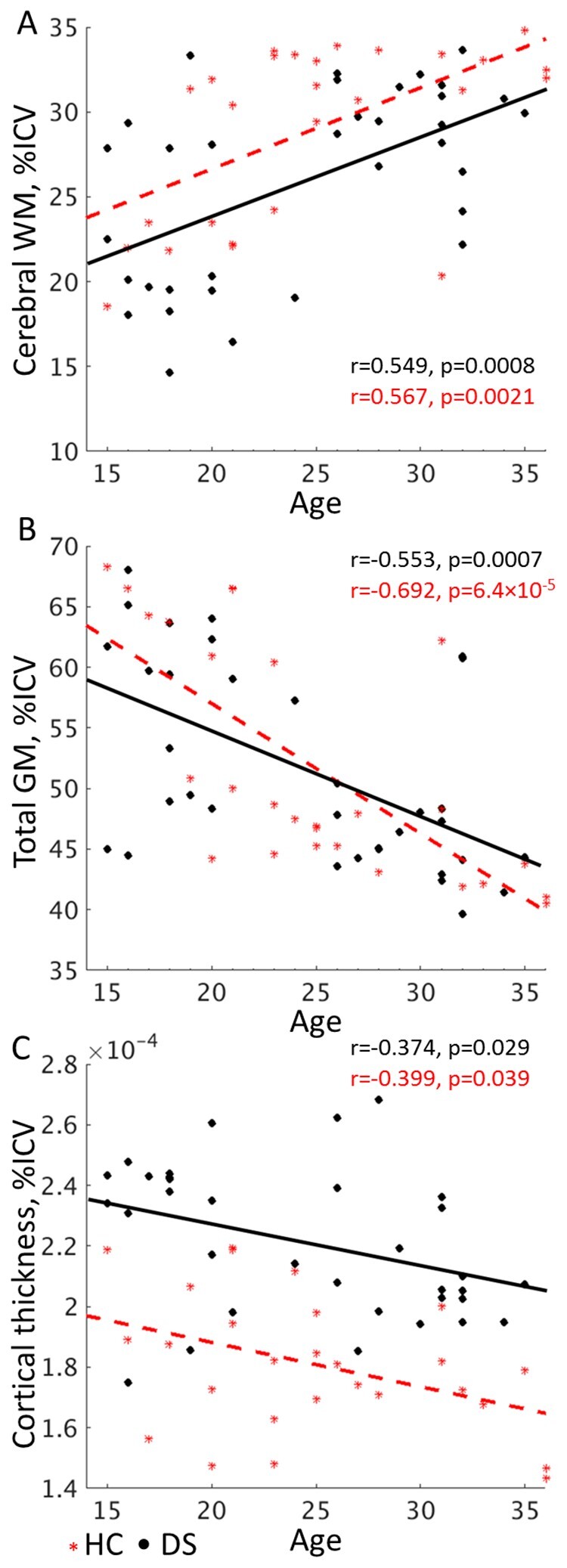
**The relationship between whole brain measures and age**. The relationship of (**A**) cerebral WM and (**B**) total GM volume and (**C**) mean cortical thickness measures to age in the control (*n* = 27) and DS (*n* = 34) groups. Although cerebral WM volume is reduced in the DS group, both groups show similar slopes, suggesting that age-related volume changes proceed similarly. DS, Down syndrome; GM, grey matter; HC, healthy control; ICV, intracranial volume; WM, white matter.

ICV was smaller in the DS group (*P *=* *3.5 × 10^−7^), and correlations between ICV and age were not significant in either group. The remainder of the results refer to ICV-corrected volumes. The DS group had increased mean cortical thickness (*P *=* *1.35 × 10^−8^) and larger mean lateral ventricle volume (*P *=* *6.09 × 10^−5^; Table 1). Cerebral WM volume was decreased in the DS group (*P *=* *0.0388), though this comparison did not survive FDR correction. There were no group differences in total or subcortical GM volumes. In both groups, age was positively related to cerebral WM volume (DS: *r* = 0.549, *P *=* *0.0008; controls: *r* = 0.567, *P *=* *0.0021; Fig. 4A), and negatively related to total GM volume (DS: *r* = −0.553, *P *=* *0.0007; controls: *r* = −0.692, *P *=* *6.4 × 10^−5^; Fig. 4B) and mean cortical thickness (DS: *r* = −0.374, *P *=* *0.029; controls: *r* = −0.399, *P *=* *0.039, Fig. 4C). Subcortical GM was related to age in controls (*r* = −0.519, *P *=* *0.0056), but not in the DS group (*P *=* *0.214). Mean lateral ventricle volume was not related to age in either group. For variables that showed a significant relationship to age, linear regression using group, age, and an age*group interaction term was used to assess group differences in the relationship between age and volume measures. No variables showed a significant age*group interaction, and results did not differ from those reported above.

**Table 1:** Group-averaged anatomical volumes and results of *t*-tests assessing group differences in ICV-corrected volumes (DS *n* = 34; HC *n* = 27)

Region	Mean volume (mm^3^)		Mean volume, % ICV		
	DS	HC	DS	HC	*P*
Whole brain					
Cerebral WM	315 131 ± 81 831	415 217 ± 94 079	26.004	28.960	0.0388
Lateral ventricle	9271 ± 3399	6909 ± 2768	0.7719	0.4896	**6.09 × 10^–5^**
Total GM	622 305 ± 123 963	735 875 ± 139 052	51.542	51.808	0.9078
Subcortical GM	40 661 ± 4919	46 818 ± 4625	3.371	3.289	0.1434
Thickness (mm)	2.64 ± 0.18	2.55 ± 0.12	2.2 **×** 10^–4^	1.8 **×** 10^–4^	**1.35 × 10^–8^**
Hippocampus, Left					
ERC	289 ± 112	368 ± 53	0.0238	0.0258	0.2068
SUB	563 ± 91	653 ± 75	0.0468	0.0459	0.5369
CA1	1021 ± 137	1446 ± 153	0.0849	0.1020	**6.6 × 10^–9^**
CAt	1048 ± 146	1496 ± 163	0.0870	0.1054	**1.9 × 10^–9^**
DG	571 ± 99	810 ± 116	0.0473	0.0569	**6.6 × 10^–7^**
Tail	110 ± 27	156 ± 27	0.0092	0.0110	**7.7 × 10^–4^**
HIP	2292 ± 298	3115 ± 319	0.1903	0.2192	**6.2 × 10^–8^**
Hippocampus, Right					
ERC	323 ± 108	371 ± 61	0.0265	0.0260	0.7666
SUB	483 ± 70	555 ± 67	0.0401	0.0391	0.4319
CA1	1220 ± 151	1614 ± 127	0.1014	0.1138	**5.2 × 10^–6^**
CAt	1230 ± 151	1636 ± 129	0.1022	0.1154	**1.6 × 10^–6^**
DG	616 ± 112	844 ± 120	0.0510	0.0593	**3.1 × 10^–5^**
Tail	86 ± 19	121 ± 26	0.0072	0.0086	**0.0025**
HIP	2415 ± 293	3156 ± 280	0.2005	0.2223	**7.6 × 10^–6^**

Bold values survived correction for multiple comparisons.

CA1, cornu Ammonis field 1; CAt, CA total; DG, dentate gyrus; DS, Down syndrome; ERC, entorhinal cortex; GM, grey matter; HC, healthy control; HIP, total hippocampus; WM, white matter; SUB, subiculum; Thickness, cortical thickness.

Measures of CA2 and CA3 were highly variable among participants, and were not analysed individually. Volumes used for analysis included left and right entorhinal cortex (ERC), SUB, CA1, CA total (CAt; the sum of CA1, CA2 and CA3), DG, tail, and total hippocampus (HIP; the sum of all included measures). Compared to controls, individuals with DS had disproportionately smaller bilateral CA1, CAt, DG, tail and HIP volumes (Table 1; Supplementary Fig. 1). Hippocampal volume measures were not significantly related to age in either group. In the group with DS, total GM was the only whole brain measure that was significantly related to any hippocampal measure—left and right ERC volumes (*r* = 0.534, *P *=* *0.0011 and *r* = 0.461, *P *=* *0.0061, respectively). These measures showed a weak relationship in controls, but did not survive FDR correction (*P *<* *0.034). In controls, mean cortical thickness was related to left and right CA1 (*r* = 0.599, *P *=* *0.0010 and *r* = 0.720, *P *=* *2.3 × 10^−5^, respectively), CAt (*r* = 0.580, *P *=* *0.0015 and *r* = 0.718, *P *=* *2.5 × 10^−5^, respectively) and HIP (*r* = 0.551, *P *=* *0.0029 and *r* = 0.659, *P *=* *0.0002, respectively). Although these measures showed positive relationships in DS, they did not survive FDR correction (*P *<* *0.124). There were no other significant relationships between hippocampal and whole-brain volumes in either group.

#### Hemispheric asymmetry

In both groups, SUB and tail volumes were larger on the left (*P *<* *7.4 × 10^−7^), and CA1, CAt, and the DG were larger on the right (*P *<* *0.0036, Table 2). In controls, left and right HIP and ERC volumes did not differ. In the DS group, HIP was larger on the right (*P *=* *0.0010), as was ERC (*P *=* *0.016), although the ERC comparison did not survive FDR. CA1 (*P *=* *0.0095) and CAt (*P *=* *0.0072) AI were larger in the DS group, indicating that the difference between left and right volumes was greater in DS compared to controls. ERC, SUB, DG, tail and HIP did not show group differences in AI.

**Table 2:** Hemispheric asymmetry of hippocampal subfield volumes (DS *n* = 34; HC *n* = 27)

Region	**Within-group** **Left versus right volumes**				**Between-group** **AI**		
	**DS**		**HC**		**DS**	**HC**	
	***P*** ^a^	**Side**	***P*** ^a^	**Side**	**Mean**	**Mean**	***P*** ^b^
ERC	0.0158	–	0.7083	–	−6.023	−0.221	0.0381
SUB	**7.4 × 10^–7^**	L	**1.2 × 10^–9^**	L	7.500	8.136	0.6933
CA1	**1.3 × 10^–10^**	R	**1.6 × 10^–10^**	R	−8.875	−5.602	**0.0095**
CAt	**1.4 × 10^–9^**	R	**2.9 × 10^–8^**	R	−8.044	−4.600	**0.0072**
DG	**0.0027**	R	**0.0036**	R	−3.702	−2.083	0.2791
Tail	**2.8 × 10^–8^**	L	**1.9 × 10^–8^**	L	11.944	12.770	0.7206
HIP	**0.0010**	R	0.1173	–	−2.652	−0.727	0.0395

AI, asymmetry index; CA1, cornu Ammonis field 1; CAt, CA total; DG, dentate gyrus; DS, Down syndrome; ERC, entorhinal cortex; HC, healthy control; HIP, total hippocampus; L, left; R, right; SUB, subiculum.

a Results of within-group paired *t*-tests showing the difference in left and right volumes, specifying the hemisphere with larger volume.

b Results of unpaired *t*-tests showing group differences in AI. Negative AI values indicates rightward asymmetry. Bold values survived correction for multiple comparisons.

### Connectivity analysis

#### Sample

The final connectivity sample included 22 individuals with DS (mean age 25.5 ± 6.5, range 15–35; 13 males) and 22 controls (mean age 25.1 ± 6.8, range 15–36; 13 males). There were no differences in age (*P *=* *0.8387) or sex (*P *=* *1.0) distribution. Of the 34 participants with DS, seven participants had no or only partial connectivity scans, and five were excluded due to motion, detailed below. In order to match the sample size and demographics of the DS group, 22 controls subjects were chosen that mostly closely matched the DS group in age and sex. Additional details can be found in the Supplementary material.

#### Motion

After preprocessing and prior to additional analysis, each time series was visually inspected for scanner or extreme motion-related artifacts, such as visible signal loss or obviously non-physiological correlation patterns. Two participants with DS were excluded from further analysis due to multiple large head movements that resulted in visible signal loss. For the remaining participants, slice-wise mean and maximum motion estimates were used to exclude participants with values greater than 2.5 standard deviations from the mean. This resulted in the removal of three additional participants with DS and no controls. Despite removal of these participants, the DS group still had higher mean (*P *=* *8.4 × 10^−5^) and max (*P *=* *0.0001) motion values compared to controls, which must be considered when interpreting the results reported below.

#### Group differences

**Figure 5:**
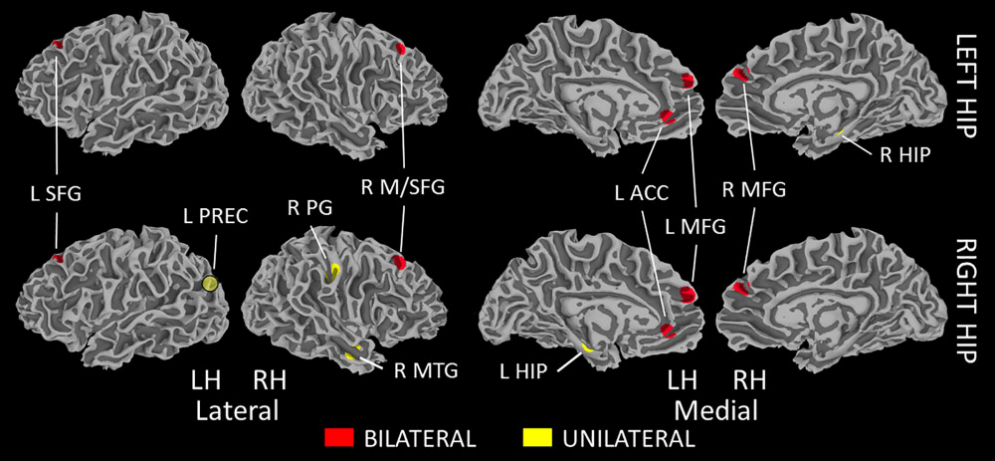
**Group differences in hippocampal connectivity. Group differences in functional connectivity measured from the left hippocampus (top) and right hippocampus (bottom; *n* = 22 in each group)**. Red denotes regions showing differences in connectivity strength to both left and right hippocampi, while yellow denotes regions showing unilateral differences. The L PREC ROI is transparent, indicating a non-surface region. All regions showed stronger connectivity in HC (*P *<* *0.0200) with the exception of L PREC, which showed stronger connectivity in DS (*P *=* *0.0090). ACC, anterior cingulate cortex; HIP, hippocampus; L, left; MFG, medial frontal gyrus; M/SFG, middle/superior frontal gyrus; MTG, middle temporal gyrus; PG, precentral gyrus; PREC, precuneus; R, right; SFG, superior frontal gyrus.

Table 3 includes all regions showing significant functional connections to the hippocampus in either group. With the exception of the connection from the right hippocampus to the left precuneus, all regions that showed significant group differences had weaker connectivity in the DS group (Fig. 5). Multiple frontal lobe regions showed lower connectivity to bilateral hippocampi in the DS group, including the bilateral medial frontal gyri (*P *<* *0.0036), the right middle/superior frontal gyrus (*P *=* *0.0039), the left superior frontal gyrus (*P *=* *0.0002), the left anterior cingulate (*P *=* *0.0002), and the right precentral gyrus (*P *=* *0.0054). Connectivity between the left and right hippocampus was also weaker in the DS group (*P *=* *0.0179), though note that this difference was not significant in the supplementary analysis assessing the impact of individual motion estimates (Supplementary Table 3). Connections were also weaker in the DS group from the right hippocampus to the right middle temporal gyrus (*P *=* *0.0050). The DS group showed a stronger connection from the right hippocampus to the left precuneus (*P *=* *0.0090). No connections showed a significant correlation to age in either group.

**Table 3:** Group differences in regions showing significant connectivity to the left and right hippocampus (*n* = 22 in both groups)

			Talairach coordinates			*P* ^a^	
Region		BA	*x*	*y*	*z*	Left	Right
L	superior frontal gyrus	8	−11	32	47	**2.4 × 10^–5^**	**0.0002**
L	middle/superior frontal gyrus	8	−21	26	46	0.1006	0.0988
R	middle/superior frontal gyrus	8	24	29	45	**0.0021**	**0.0039**
L	medial frontal gyrus	9	−3	49	28	**0.0036**	**0.0001**
R	medial frontal gyrus	9	5	49	26	**0.0009**	**4.2 × 10^–5^**
R	precentral gyrus	4	56	−13	38	0.0867	**0.0054**
L	anterior cingulate	24	−4	37	1	**0.0002**	**4.3 × 10^–5^**
L	posterior cingulate	30	−6	−50	17	0.6676	0.7268
R	posterior cingulate	31	5	−50	18	0.3129	0.3916
L	middle temporal gyrus	21	−57	−3	−13	0.0566	0.1751
R	middle temporal gyrus	21	52	−1	−20	0.0312	**0.0050**
L	superior/middle temporal gyrus	39	−47	−65	32	0.6163	0.7120
R	superior/middle temporal gyrus	39	46	−64	24	0.8190	0.3507
L	lingual gyrus	17	−4	−82	0	0.0241	0.0763
R	lingual gyrus	17	4	−78	5	0.1154	0.0589
L	Precuneus	7	−27	−69	33	0.1205	**0.0090** ^b^
L	Hippocampus		24	−15	−14	–	**0.0073**
R	Hippocampus		−24	−16	−10	**0.0179**	–
L	parahippocampal gyrus	35	−22	−35	−7	0.9267	0.5625
R	parahippocampal gyrus	35	22	−34	−11	0.8038	0.8259

BA, Brodmann area; L, left; R, right.

a Results of Student's *t*-tests between groups. Values in bold survived correction for multiple comparisons.

b Regions where connectivity is stronger in DS as compared to controls.

#### Hemispheric asymmetry

In the control group, connectivity to the left middle temporal gyrus was stronger from the left than from the right hippocampus (*P *=* *0.0015). The DS group did not show differences in the strength of connectivity from the right or left hippocampus.

#### Relationship to hippocampal volumes

In the DS group, inter-hemispheric hippocampal connectivity was significantly related to CA1 and CAt volumes. Connectivity from the left to the right hippocampus was related to left CA1 (*r* = 0.641, *P *=* *0.0013) and CAt (*r* = 0.607, *P *=* *0.0027) volumes. Connectivity from the right to the left hippocampus was related to right CA1 (*r* = 0.604, *P *=* *0.0029) and CAt (*r* = 0.596, *P *=* *0.0034) volumes. In the control group, connectivity from the left hippocampus to the bilateral medial frontal gyri was positively related to bilateral HIP (*P *<* *0.0383), but these relationships did not survive FDR correction. No other connections were related to hippocampal volumes in either group.

## Discussion

This work used high resolution, 7T MRI to assess structure and function of the hippocampus in individuals with DS. The gains in signal-to-noise ratio, tissue contrast and spatial resolution achievable at 7T provide a level of detail and accuracy that has not previously been attainable in in-vivo studies of individuals with DS. In addition to increased whole-brain mean cortical thickness and lateral ventricle volumes, hippocampal subfield volumes showed selective reductions in bilateral volumes of CA1, DG, and tail in the DS group, but no differences in ERC or SUB volumes. In the DS group, bilateral hippocampi showed widespread decreases in the strength of connectivity, predominately to frontal regions. Both groups showed similar relationships of age to whole-brain volume measures, but not to hippocampal volumes or connectivity.

Our results are consistent with reports of reduced hippocampal volume in individuals with DS compared to controls.^11,12,51–54^ The few investigations of hippocampal subfields in DS used T21 foetal brain tissue, and while not directly comparable to adult brains, provide some comparison for our findings. Our finding of reduced DG volume in DS is consistent with reports of fewer myelinated axons,^21^ decreased volume,^19^ and reduced cell proliferation^20^ in T21 foetal brains. The finding of decreased volume of CA1, but not the SUB, is surprising in light of the reported involvement of these regions in both DS and Alzheimer’s disease pathology. In T21 foetal brains, CA1 and the SUB are reported to show decreased volumes^18^^,^^19^ and increased phagocytic activity.^17^ In non-DS Alzheimer’s disease and MCI, CA1 and the SUB are the earliest hippocampal regions to show pathology, and the SUB, presubiculum, and tail showed reductions in a sample of elderly individuals with amyloid-β pathology but no signs of cognitive decline.^26^ Perhaps less surprising is the lack of difference in ERC volume seen in the present sample, given reports of increased volume of the parahippocampal gyrus in DS.^11^^,^^12^^,^^54^ The hippocampus shows reliable hemispheric asymmetry in the general population, with larger volume on the right.^55^ Our findings of rightward asymmetry in CA1, CAt and DG agree with those of a report of subfield lateralization in 100 healthy controls.^56^ However, our sample showed an increase in SUB volume to the left that was not found in the larger study, which contributed to the lack of lateralization of overall hippocampal volume reported in our control group. Increased AI of CA regions in the DS group is notable in light of increased asymmetry of the hippocampus in non-DS Alzheimer’s disease and MCI,^56^ although subfields showing some of the largest AI differences in that report (the SUB and DG) were not significant here.

Whole-brain anatomical measures were not the focus of this investigation, but were reported for completeness. Our findings support reports of reduced intracranial and increased ventricular volumes in individuals with DS.^11^^,^^53^^,^^54^^,^^57^ While cerebral WM volume showed a non-significant reduction in the DS group, cortical and subcortical GM volumes were not significantly different. Previous investigations of brain volumes in individuals with DS often focussed on regional rather than whole-brain measures, and have reported volume increases and decreases.^11^^,^^54^ This may explain why the coarser whole-brain measures reported here did not show group differences. We did find increased cortical thickness in the DS group, in agreement with previous reports,^58^^,^^59^ although we did not account for the impact of grey–white matter contrast, previously reported as altered in DS.^60^ Of note, both groups showed relationships of a similar magnitude between age and increased WM and decreased cortical GM. These relationships are in line with reported age-related volume changes in the general population,^61^^,^^62^ and suggest that, despite potential differences in baseline level, whole-brain volume measures in young, non-demented individuals with DS may follow a similar trajectory as those in the general population.

The hippocampus has widespread cortical connections, including strong reciprocal connections to the PCC through the cingulum bundle.^63^ Deep brain stimulation of the PCC has been shown to modulate hippocampal activity and impair episodic memory performance,^64^ and the integrity of posterior cingulum bundle WM has been related to memory and executive function in patients with early Alzheimer’s disease.^65^ These findings, along with reports of disrupted functional connectivity between the hippocampus and PCC in Alzheimer’s disease and MCI^30^^,^^33^^,^^34^ and the strong functional connections between the head of the hippocampus and regions comprising the DMN,^66^ led us to focus on the hippocampal–PCC relationship for creation of seed ROIs. Indeed, the resulting connectivity maps showed substantial overlap with regions considered to be part of the DMN.^67^ Reports of decreased functional connectivity of the DMN in preclinical and clinical Alzheimer’s disease,^35^^,^^36^ and of decreased global brain connectivity to the PCC in DS,^37^ suggested that hippocampal connectivity may also be reduced in the DS group.

Previous studies of functional connectivity in DS report widespread changes. Studies reporting between-network connectivity have generally found increased connection strength in those with DS,^68^^,^^69^ suggesting disrupted between-network interactions. Studies assessing regional connectivity have returned mixed results. Wilson *et al*.^70^ found decreased connectivity strength to the medial frontal gyrus in individuals with DS, with more reductions in older individuals with positive amyloid PET scans. Pujol *et al*.^37^ found that individuals with DS showed both increased and decreased regional connectivity compared to controls, and connectivity strength showed both positive and negative relationships to behavioural measures. These findings suggest that both increased and decreased synchrony contribute to cognitive dysfunction in DS, with directionality likely dependent on regional relationships and network dynamics. By limiting our analysis to regions that were synchronous with the hippocampus in either group, we focussed on within-network connectivity. We did not find group differences in our primary measure of interest, hippocampal-PCC connectivity. Instead, we found bilateral reductions in connectivity to frontal regions and to the contralateral hippocampus in the DS group. Only one region showed increased connectivity in the DS group, that of the left precuneus to the right hippocampus. That most of the changes were in the frontal lobe and anterior temporal regions is in line with Pujol *et al*.,^38^ who found increased connectivity in DS in ventral regions of the frontal and temporal lobes and decreased connectivity in dorsal regions. The suggestion of regionally specific changes may explain an inconsistency between the current results and that of a recent study in the Ts65Dn mouse model of DS. Compared to control animals, neural activity measured in the Ts65Dn mice showed increased phase synchronization of the prefrontal cortex and hippocampus during rest at multiple frequencies. It is possible that the opposite relationship found in our study is related to the functional and anatomical specificity achievable with high-field MRI, allowing us to focus on discrete regions of the frontal lobe.

Head motion is an issue for all MRI studies, and leads to measurable changes in connectivity.^71^ Previous studies of functional connectivity in DS have dealt with motion in various ways, including censoring volumes with large motion estimates^68^ and using motion estimates as regressors in group analysis.^37^^,^^69^ The motion correction algorithm used here accounts for both volumetric and slice-specific motion, resulting in more accurate motion correction than methods that account for volumetric motion alone.^45^ A number of factors increase our confidence in the accuracy of our results, including the finding of both increased and decreased connectivity in the DS group, highly consistent and symmetric findings from both hippocampi, and significant relationships with volumetric measures. Additionally, a supplementary analysis showed that accounting for individual residual motion estimates did not lead to substantial changes in our results. Still, as with all studies showing group differences in motion level, our results should be interpreted with caution.

Previous studies have reported selective deficits in hippocampally-mediated cognitive functions in individuals with DS.^9^^,^^72^ Of note, we found an intriguing association between hippocampally-mediated long term visual memory performance and the volume of CA1. Reduced CA1 volume may reflect changes in cell function, number, and/or structure, impacting cognitive performance. Note that our sample includes only participants who were able to successfully complete an MRI, which results in a bias towards higher levels of functioning. In addition to further histological work, longitudinal measures, ideally in a sample including individuals across the range of function, will be required to describe the interplay between volumes and cognitive performance.

Using high-resolution imaging, the present work confirms smaller hippocampal volumes in individuals with DS. To our knowledge, this is the first in-vivo comparison of hippocampal subfield volumes between individuals with DS and typically-developing controls. We found preferential decreases in CA regions, DG, and tail. Further, individuals with DS show reductions in functional connectivity of the hippocampus, primarily to frontal lobe regions, with increased connectivity between the hippocampus and precuneus. We also found significant relationships between volumetric and cognitive measures. Although future work to validate these exploratory findings will be necessary, these results support the investigation of specific MRI-derived measures as surrogate markers of drug efficacy in pharmacological studies designed to investigate the possible enhancement of cognitive function in persons with DS. Lastly, the finding that neither hippocampal volume nor functional connectivity changes were associated with age in this sample of teenagers and young adults with DS is important from the perspective of studying neurodegenerative processes and their potential prevention in those with DS. As even young individuals with DS can display Alzheimer’s disease-like neuropathology, this finding points toward a window of time during which, although pathology may be present, it may exist in the context of well-preserved neural structure and function, i.e. in a state in which potential therapeutic interventions would have their best chance of being effective.

## Supplementary material

Supplementary material is available at *Brain Communications* online.

## Supplementary Material

fcab088_Supplementary_Data

## Abbreviations

AI =: asymmetry index
ASHS =: Automated Segmentation of Hippocampal Subfields
CA1 =: cornu Ammonis field 1
CAt =: CA total
DG =: dentate gyrus
DMN =: default mode network
DS =: Down syndrome
ERC =: entorhinal cortex
FDR =: false discovery rate
GM =: grey matter
HC =: healthy control
HIP =: total hippocampus
ICV =: intracranial volume
MCI =: mild cognitive impairment
PCC =: posterior cingulate cortex
ROI =: region of interest
SUB =: subiculum
T21 =: trisomy of chromosome 21
WM =: white matter

## References

[1] Roizen NJ, Patterson D. Down’s syndrome. Lancet. 2003;361(9365):1281–1289. 12699967 10.1016/S0140-6736(03)12987-X

[2] Capone GT, Chicoine B, Bulova P, et al Down Syndrome Medical Interest Group DSMIG-USA Adult Health Care Workgroup. Co-occurring medical conditions in adults with Down syndrome: A systematic review toward the development of health care guidelines. Am J Med Genet A. 2018;176(1):116–133. 29130597 10.1002/ajmg.a.38512

[3] Capone G, Stephens M, Santoro S, et al Down Syndrome Medical Interest Group (DSMIG‐USA) Adult Health Workgroup. Co-occurring medical conditions in adults with Down syndrome: A systematic review toward the development of health care guidelines part II. Am J Med Genet A. 2020;182(7):1832–1814. 32338447 10.1002/ajmg.a.61604

[4] Hamburg S, Lowe B, Startin CM, et al Assessing general cognitive and adaptive abilities in adults with Down syndrome: A systematic review. J Neurodev Disord. 2019;11(1):1–16. 31470792 10.1186/s11689-019-9279-8 PMC6716931

[5] Nelson L, Johnson JK, Freedman M, et al Learning and memory as a function of age in Down syndrome: A study using animal-based tasks. Prog Neuropsychopharmacol Biol Psychiatry. 2005;29(3):443–453. 15795053 10.1016/j.pnpbp.2004.12.009

[6] Abbeduto L, Murphy MM, Cawthon SW, et al Receptive language skills of adolescents and young adults with Down or Fragile X syndrome. Am J Ment Retard. 2003;108(3):149–160. 12691594 10.1352/0895-8017(2003)108<0149:RLSOAA>2.0.CO;2

[7] Jarrold C, Baddeley AD, Hewes AK. Verbal short-term memory deficits in Down syndrome: A consequence of problems in rehearsal? J Child Psychol Psychiatry. 2000;41(2):233–244. 10750549

[8] Carlesimo GA, Marotta L, Vicari S. Long-term memory in mental retardation: Evidence for a specific impairment in subjects with Down's syndrome. Neuropsychologia. 1997;35(1):71–79. 8981379 10.1016/s0028-3932(96)00055-3

[9] Pennington BF, Moon J, Edgin J, Stedron J, Nadel L. The neuropsychology of Down syndrome: Evidence for hippocampal dysfunction. Child Dev. 2003;74(1):75–93. 12625437 10.1111/1467-8624.00522

[10] Kates WR, Folley BS, Lanham DC, Capone GT, Kaufmann WE. Cerebral growth in Fragile X syndrome: Review and comparison with Down syndrome. Microsc Res Tech. 2002;57(3):159–167. 12112452 10.1002/jemt.10068

[11] Raz N, Torres IJ, Briggs SD, et al Selective neuroanatomic abnormalities in Down’s syndrome and their cognitive correlates: Evidence from MRI morphology. Neurology. 1995;45(2):356–366. 7854539 10.1212/wnl.45.2.356

[12] Kesslak JP, Nagata SF, Lott IT, Nalcioglu O. MRI analysis of age-related changes in the brains of individuals with DS. Neurology. 1994;44(6):1039–1045. 8208396 10.1212/wnl.44.6.1039

[13] Teipel SJ, Alexander GE, Schapiro MB, Möller H‐J, Rapoport SI, Hampel H. Age-related cortical grey matter reductions in non-demented Down's sydrome adults determined by MRI with voxel-based morphometry. Brain. 2004;127(4):811–824. 14985261 10.1093/brain/awh101

[14] Śmigielska-Kuzia J, Boćkowski L, Sobaniec W, et al A volumetric magnetic resonance imaging study of brain structures in children with Down syndrome. Neurol Neurochir Pol. 2011;45(4):363–369. 22101997 10.1016/s0028-3843(14)60107-9

[15] Eichenbaum H, Yonelinas AR, Ranganath C. The medial temporal lobe and recognition memory. Annu Rev Neurosci. 2007;30(1):123–152. 17417939 10.1146/annurev.neuro.30.051606.094328 PMC2064941

[16] Bird CM, Burgess N. The hippocampus and memory: Insights from spatial processing. Nat Rev Neurosci. 2008;9(3):182–194. 18270514 10.1038/nrn2335

[17] Kanaumi T, Milenkovic I, Adle-Biassette H, Aronica E, Kovacs GG. Non-neuronal cell responses differ between normal and Down syndrome developing brains. Int J Dev Neurosci. 2013;31(8):796–803. 24113258 10.1016/j.ijdevneu.2013.09.011

[18] Stagni F, Giacomini A, Emili M, et al Subicular hypotrophy in fetuses with Down syndrome and in the Ts65Dn model of Down syndrome. Brain Pathol. 2019;29(3):366–379. 30325080 10.1111/bpa.12663 PMC8028278

[19] Guidi S, Bonasoni P, Ceccarelli C, et al Neurogenesis impairment and increased cell death reduce total neuron number in the hippocampal region of fetuses with Down syndrome. Brain Pathol. 2007;18(2):180–197. 18093248 10.1111/j.1750-3639.2007.00113.x PMC8095525

[20] Contestabile A, Fila T, Ceccarelli C, et al Cell cycle alteration and decreased cell proliferation in the hippocampal dentate gyrus and in the neocortical germinal matrix of fetuses with Down syndrome and in Ts65Dn mice. Hippocampus. 2007;17(8):665–678. 17546680 10.1002/hipo.20308

[21] Ábrahám H, Vincze A, Veszprémi B, et al Impaired myelination of the human hippocampal formation in Down syndrome. Int J Dev Neurosci. 2012;30(2):147–158. 22155002 10.1016/j.ijdevneu.2011.11.005

[22] Pujol J, Fenoll R, Ribas-Vidal N, et al A longitudinal study of brain anatomy changes preceding dementia in Down syndrome. Neuroimage Clin. 2018;18:160–166. 29868444 10.1016/j.nicl.2018.01.024 PMC5984600

[23] Beacher F, Daly E, Simmons A, et al Alzheimer’s disease and Down’s syndrome: An in vivo MRI study. Psychol Med. 2009;39(4):675–684. 18667098 10.1017/S0033291708004054

[24] de Flores R, La Joie R, Chételat G. Structural imaging of hippocampal subfields in healthy aging and Alzheimer's disease. Neuroscience. 2015;309:29–50. 26306871 10.1016/j.neuroscience.2015.08.033

[25] Apostolova LG, Mosconi L, Thompson PM, et al Subregional hippocampal atrophy predicts Alzheimer's dementia in the cognitively normal. Neurobiol Aging. 2010;31(7):1077–1088. 18814937 10.1016/j.neurobiolaging.2008.08.008 PMC2873083

[26] Hsu PJ, Shou H, Benzinger T, et al Amyloid burden in cognitively normal elderly is associated with preferential hippocampal subfield volume loss. J Alzheimers Dis. 2015;45(1):27–33. 25428255 10.3233/JAD-141743 PMC4351157

[27] Milenkovic I, Stojanovic T, Aronica E, et al GABA_A_ receptor subunit deregulation in the hippocampus of human foetuses with Down syndrome. Brain Struct Funct. 2018;223(3):1501–1518. 29168008 10.1007/s00429-017-1563-3 PMC5869939

[28] Leverenz JB, Raskind MA. Early amyloid deposition in the medial temporal lobe of young Down syndrome patients: A regional quantitative analysis. Exp Neurol. 1998;150(2):296–304. 9527899 10.1006/exnr.1997.6777

[29] Davidson YS, Robinson A, Prasher VP, Mann DMA. The age of onset and evolution of Braak tangle stage and Thal amyloid pathology of Alzheimer's disease in individuals with Down syndrome. Acta Neuropathol Commun. 2018;6(1):1–11. 29973279 10.1186/s40478-018-0559-4 PMC6030772

[30] Wang L, Zang Y, He Y, et al Changes in hippocampal connectivity in the early stages of Alzheimer's disease: Evidence from resting state fMRI. NeuroImage. 2006;31(2):496–504. 16473024 10.1016/j.neuroimage.2005.12.033

[31] Xue J, Guo H, Gao Y, et al Altered directed functional connectivity of the hippocampus in mild cognitive impairment and Alzheimer's disease: A resting-state fMRI study. Front Aging Neurosci. 2019;11(326):1–15. 31866850 10.3389/fnagi.2019.00326 PMC6905409

[32] Zarei M, Beckmann CF, Binnewijzend MA, et al Functional segmentation of the hippocampus in the healthy human brain and in Alzheimer's disease. NeuroImage. 2013;66:28–35. 23128076 10.1016/j.neuroimage.2012.10.071

[33] Dunn CJ, Duffy SL, Hickie IB, et al Deficits in episodic memory retrieval reveal impaired default mode network connectivity in amnestic mild cognitive impairment. Neuroimage Clin. 2014;4:473–480. 24634833 10.1016/j.nicl.2014.02.010 PMC3952352

[34] Zhou Y, Dougherty JH, Hubner KF, Bai B, Cannon RL, Hutson RK. Abnormal connectivity in the posterior cingulate and hippocampus in early Alzheimer's disease and mild cognitive impairment. Alzheimers Dement. 2008;4(4):265–270. 18631977 10.1016/j.jalz.2008.04.006

[35] Palmqvist S, Schöll M, Strandberg O, et al Earliest accumulation of β-amyloid occurs within the default-mode network and concurrently affects brain connectivity. Nat Commun. 2017;8(1):1214. 29089479 10.1038/s41467-017-01150-x PMC5663717

[36] Wu X, Li R, Fleisher AS, et al Altered default mode network connectivity in Alzheimer's disease – A resting functional MRI and Bayesian network study. Hum Brain Mapp. 2011;32(11):1868–1881. 21259382 10.1002/hbm.21153 PMC3208821

[37] Pujol J, del Hoyo L, Blanco-Hinojo L, et al Anomalous brain functional connectivity contributing to poor adaptive behavior in Down syndrome. Cortex. 2015;64:148–156. 25461715 10.1016/j.cortex.2014.10.012

[38] Alemany-González M, Gener T, Nebot P, Vilademunt M, Dierssen M, Puig MV. Prefrontal–hippocampal functional connectivity encodes recognition memory and is impaired in intellectual disability. PNAS. 2020;117(21):11788–11798. 32393630 10.1073/pnas.1921314117 PMC7261130

[39] Basten IA, Boada R, Taylor HG, et al On the design of broad-based neuropsychological test batteries to assess the cognitive abilities of individuals with Down syndrome in the context of clinical trials. Brain Sci. 2018;8(12):205. 10.3390/brainsci8120205 PMC6315396 30486228

[40] Dale AM, Fischl B, Sereno MI. Cortical surface-based analysis. I. Segmentation and surface reconstruction. NeuroImage. 1999;9(2):179–194. 9931268 10.1006/nimg.1998.0395

[41] Yushkevich PA, Pluta J, Wang H, et al Automated volumetry and regional thickness analysis of hippocampal subfields and medial temporal cortical structures in mild cognitive impairment. Hum Brain Mapp. 2015;36(1):258–287. 25181316 10.1002/hbm.22627 PMC4313574

[42] Wisse LEM, Kuijf HJ, Honingh AM, et al Automated hippocampal subfield segmentation at 7T MRI. Am J Neuroradiol. 2016;37(6):1050–1057. 26846925 10.3174/ajnr.A4659 PMC4907820

[43] Benjamini Y, Hochberg Y. On the adaptive control of the false discovery rate in multiple testing with independent statistics. J Educ Behav Stat. 2000;25(1):60–83.

[44] Glover GH, Li T, Ress D. Image-based method for retrospective correction of physiological motion effects in fMRI: RETROICOR. Magn Reson Med. 2000;44(1):162–167. 10893535 10.1002/1522-2594(200007)44:1<162::aid-mrm23>3.0.co;2-e

[45] Beall EB, Lowe MJ. SimPACE: Generating simulated motion corrupted BOLD data with synthetic-navigated acquisition for the development and evaluation of SLOMOCO: A new, highly effective slicewise motion correction. NeuroImage. 2014;101:21–34. 24969568 10.1016/j.neuroimage.2014.06.038 PMC4165749

[46] Cox R. AFNI: Software for analysis and visualization of functional magnetic resonance neuroimages. Comput Biomed Res. 1996;29(3):162–173. 8812068 10.1006/cbmr.1996.0014

[47] Saad ZS, Glen DR, Chen G, Beauchamp MS, Desai R, Cox RW. A new method for improving functional-to-structural alignment using local Pearson correlation. NeuroImage. 2009;44(3):839–848. 18976717 10.1016/j.neuroimage.2008.09.037 PMC2649831

[48] Poppenk J, Moscovitch M. A hippocampal marker of recollection memory ability among healthy young adults: Contributions of posterior and anterior segments. Neuron. 2011;72(6):931–937. 22196329 10.1016/j.neuron.2011.10.014

[49] Yeo BT, Krienen FM, Sepulcre J, et al The organization of the human cerebral cortex estimated by intrinsic functional connectivity. J Neurophysiol. 2011;106(3):1125–1165. 21653723 10.1152/jn.00338.2011 PMC3174820

[50] Lowe MJ, Mock BJ, Sorenson JA. Functional connectivity in single and multislice echoplanar imaging using resting-state fluctuations. NeuroImage. 1998;7(2):119–132. 9558644 10.1006/nimg.1997.0315

[51] Carducci F, Onorati P, Condoluci C, et al Whole-brain voxel-based morphometry study of children and adolescents with Down syndrome. Funct Neurol. 2013;28(1):19–28. 23731912 PMC3812718

[52] Teipel SJ, Schapiro MB, Alexander GE, et al Relation of corpus callosum and hippocampal size to age in nondemented adults with Down’s syndrome. Am J Psychiatry. 2003;160(10):1870–1878. 14514503 10.1176/appi.ajp.160.10.1870

[53] Aylward EH, Li Q, Honeycutt NA, et al MRI volumes of the hippocampus and amygdala in adults with Down’s syndrome with and without dementia. Am J Psychiatry. 1999;156:564–568. 10200735 10.1176/ajp.156.4.564

[54] White NS, Alkire MT, Haier RJ. A voxel-based morphometric study of nondemented adults with Down syndrome. NeuroImage. 2003;20(1):393–403. 14527599 10.1016/s1053-8119(03)00273-8

[55] Pedraza O, Bowers D, Gilmore R. Asymmetry of the hippocampus and amygdala in MRI volumetric measurements of normal adults. J Int Neuropsychol Soc. 2004;10(5):664–678. 15327714 10.1017/S1355617704105080

[56] Sarica A, Vasta R, Novellino F, et al The Alzheimer's Disease Neuroimaging Initiative. MRI asymmetry index of hippocampal subfields increases through the continuum from the mild cognitive impairment to the Alzheimer's disease. Front Neurosci. 2018;12:576. 30186103 10.3389/fnins.2018.00576 PMC6111896

[57] Beacher F, Daly E, Simmons A, et al Brain anatomy and ageing in non-demented adults with Down's syndrome: An in vivo MRI study. Psychol Med. 2010;40(4):611–629. 19671216 10.1017/S0033291709990985

[58] Levman J, MacDonald A, Baumer N, et al Structural magnetic resonance imaging demonstrates abnormal cortical thickness in Down syndrome: Newborns to young adults. Neuroimage Clin. 2019;23:101874–101878. 31176294 10.1016/j.nicl.2019.101874 PMC6551568

[59] Lee NR, Adeyemi EI, Lin A, et al Dissociations in cortical morphometry in youth with Down syndrome: Evidence for reduced surface area but increased thickness. Cereb Cortex. 2016;26(7):2982–2990. 26088974 10.1093/cercor/bhv107 PMC4898663

[60] Bletsch A, Mann C, Andrews DS, et al Down syndrome is accompanied by significantly reduced cortical grey–white matter tissue contrast. Hum Brain Mapp. 2018;39(10):4043–4054. 29885016 10.1002/hbm.24230 PMC6866483

[61] Mills KL, Goddings A, Herting MM, et al Structural brain development between childhood and adulthood: Convergence across four longitudinal samples. NeuroImage. 2016;141:273–281. 27453157 10.1016/j.neuroimage.2016.07.044 PMC5035135

[62] Coupé P, Catheline G, Lanuza E, Manjón JV, Initiative ADN., Alzheimer's Disease Neuroimaging Initiative. Towards a unified analysis of brain maturation and aging across the entire lifespan: A MRI analysis. Hum Brain Mapp. 2017;38(11):5501–5518. 28737295 10.1002/hbm.23743 PMC6866824

[63] Wakana S, Jiang H, Nagae-Poetscher LM, van Zijl PC, Mori S. Fiber tract-based atlas of human white matter anatomy. Radiology. 2004;230(1):77–87. 14645885 10.1148/radiol.2301021640

[64] Natu VS, Lin J, Burks A, Arora A, Rugg MD, Lega B. Stimulation of the posterior cingulate cortex impairs episodic memory encoding. J Neurosci. 2019;39(36):7173–7182. 31358651 10.1523/JNEUROSCI.0698-19.2019 PMC6733540

[65] Lin Y-C, Shih Y-C, Tseng W-YI, et al Cingulum correlates of cognitive functions in patients with mild cognitive impairment and early Alzheimer’s disease: A diffusion spectrum imaging study. Brain Topogr. 2014;27(3):393–402. 24414091 10.1007/s10548-013-0346-2

[66] Zhong Q, Xu H, Qin J, Zeng L-L, Hu D, Shen H. Functional parcellation of the hippocampus from resting-state dynamic functional connectivity. Brain Res. 2019;1715:165–175. 30910629 10.1016/j.brainres.2019.03.023

[67] Horn A, Ostwald D, Reisert M, Blankenburg F. The structural‐functional connectome and the default mode network of the human brain. NeuroImage. 2014;102(1):142–151. 24099851 10.1016/j.neuroimage.2013.09.069

[68] Anderson JS, Nielsen JA, Ferguson MA, et al Abnormal brain synchrony in Down syndrome. NeuroImage Clin. 2013;2:703–715. 24179822 10.1016/j.nicl.2013.05.006 PMC3778249

[69] Vega JN, Hohman TJ, Pryweller JR, Dykens EM, Thornton-Wells TA. Resting-state functional connectivity in individuals with Down syndrome and Williams syndrome compared with typically developing controls. Brain Connectivity. 2015;5(8):461–475. 25712025 10.1089/brain.2014.0266 PMC4601631

[70] Wilson LR, Vatansever D, Annus T, et al Differential effects of Down's syndrome and Alzheimer's neuropathology on default mode connectivity. Hum Brain Mapp. 2019;40(15):4551–4563. 31350817 10.1002/hbm.24720 PMC6865660

[71] Van Dijk KRA, Sabuncu MR, Buckner RL. The influence of head motion on intrinsic functional connectivity MRI. NeuroImage. 2012;59(1):431–438. 21810475 10.1016/j.neuroimage.2011.07.044 PMC3683830

[72] Clark CAC, Fernandez F, Sakhon S, Spanò G, Edgin JO. The medial temporal memory system in Down syndrome: Translating animal models of hippocampal compromise. Hippocampus. 2017;27(6):683–691. 28346765 10.1002/hipo.22724 PMC8109260

