# Supplementary material for: High resolution structural and functional MRI of the hippocampus in young adults with Down syndrome
Source: Brain Commun. 2021 Apr 19;3(2):fcab088. doi: 10.1093/braincomms/fcab088 (PMC8100000; doi:10.1093/braincomms/fcab088)

Supplementary Materials

Participants

Data for participants with DS was collected from 06/2015 to 12/2019. Data for controls was collected from 06/2015 to 11/2019. There was no overlap of participants between the three protocols.

Protocol 1 was an observational study of adults with DS. Under Protocol 1, 16 individuals with DS were enrolled and scanned (mean age 29.8 ± 2.8; 11 males). The most common co-morbidities in this sample included hypothyroidism (n = 11), obstructive sleep apnea (n = 4), hearing loss (n = 4), and congenital heart defect (n = 3).

Protocol 2 was a clinical trial of memantine in teenagers and young adults with DS. Only baseline scans, acquired prior to any research intervention, were used in this analysis. Under Protocol 2, 26 individuals with DS (mean age 19.7 ± 4.7; 16 males) were enrolled. Five of those individuals did not complete any MRI scans (mean age 20.6 ± 4.9; 4 males). Of the five, three were not able to tolerate the scanner environment and aborted the session prior to any scanning. The remaining two were unable to stay still in the scanner, and the session was aborted prior to the completion of the first scan. These five individuals did not show age or sex differences from the remaining sample, but did show lower scores on the CVLT Total (12.2 vs. 5.4, $p = 0.031$), CVLT Recall (0.70 vs. -0.24, $p = 0.020$) and TROG-2 (19.9 vs. 14.2, $p = 0.034$). Participants that were scanned under Protocol 2 included the remaining 21 individuals with DS (mean age 19.5 ± 4.8; 12 males) and 12 controls (mean age 20.2 ± 4.1; 6 males). In the sample with DS, the most common co-morbidities included hypothyroidism (n = 10), obstructive sleep apnea (n = 6), hearing loss (n = 10), and congenital heart defect (n = 7).

Protocol 3 was an observational study of epilepsy. Additional healthy controls were selected from Protocol 3 in order to create an age- and sex-matched control group. Protocol 3 enrolled 50 controls (mean age 30.5 ± 9.5; 27 males), 15 of which were chosen for inclusion in the current analysis (mean age 28.6 ± 4.7; 11 males). Selection of controls from Protocol 3 was based entirely on age and sex matching and occurred prior to data analysis. Healthy controls included in Protocols 2 and 3 were recruited from the same geographic area.

Volumetric analysis sample

All participants from Protocols 1 and 3, and all controls from Protocol 2, were included in the volumetric analysis. Three participants with DS from Protocol 2 did not have MP2RAGE scans of sufficient quality to run using the ASHS software, and were excluded from further analysis (ages 22, 17, and 15 years, all males). The excluded participants did not differ in age, sex, or cognitive test scores from the remainder of the participants with DS from Protocol 2 (n = 18; mean age 19.7 ± 5.0; 11 males).

Connectivity analysis sample

Of the 16 participants with DS included in Protocol 1, three did not have connectivity scans (ages 34, 26, and 31 years, all males). One additional participant was removed due to motion (age 31, male). These participants did not differ in age or sex from the included participants from Protocol 1 (n = 12; mean age 29.6 ± 2.7; 7 males).

Of the 18 remaining participants with DS from Protocol 2, four did not have connectivity scans (mean age 19.8 ± 3.1; 3 males). Four additional participants were removed due to motion (mean age 17.5 ± 2.6; 2 males). Neither group differed in age, sex, or cognitive scores from the included participants with DS from Protocol 2 (n = 10; mean age 20.6 ± 6.2; 6 males).

Of the 22 controls included in the connectivity analysis, 11 were from Protocol 2 (mean age 19.9 ± 4.2; 5 males) and 11 were from Protocol 3 (mean age 30.3 ± 4.5; 8 males). These controls were selected from the sample included in the volumetric analysis. Selection was based entirely on age and sex matching and occurred prior to data analysis.

Cognitive data analysis

Cognitive testing

Individuals with DS who participated under Protocol 2 underwent neuropsychological testing. Supplementary Table 1 shows cognitive scores for participants included in the volumetric and connectivity analyses. The following measures were used in an exploratory analysis of the relationship of cognition to anatomical variables (measure - cognitive domain; variables):

1. California Verbal Learning Test-II (CVLT) Short Form – Verbal episodic memory; immediate recall total correct, free recall discrimination.
2. Differential Ability Scales (DAS), Recall of Digits Forward – Verbal short term memory; digits forward total.
3. Cambridge Neuropsychological Test Automated Battery (CANTAB); Paired Associates Learning (PAL) – Long-term visual episodic memory; first trial total correct.
4. CANTAB; Pattern Recognition Memory (PRM) – Long-term visual episodic memory; total correct, immediate correct, delayed correct.
5. DAS, Matrices – Non-verbal reasoning ability; ability score.
6. Test for Reception of Grammar II (TROG) – Syntactic comprehension; total correct.
7. Peabody Picture Vocabulary Test IV (PPVT) – Receptive semantics; raw score.

8. CANTAB; Spatial Working Memory (SWM) – Spatial working memory; strategy score, total errors.
9. Go/No-Go Task – Inhibitory control; Go reaction time.
10. CANTAB; Spatial Span (SSP) – Visual short term memory; span length.

Statistical analysis

Linear correlations assessed the relationship of anatomical volumes to cognitive scores for the 18 DS participants with both measures. The FDR was applied. Due to the small number of participants with both cognitive scores and connectivity measures (n = 10), only the volumetric analysis is reported.

Results

The complete results of the correlation analysis can be found in Supplementary Table 2, and significant relationships are shown in Supplementary Figure 2. PRM total correct and PRM delayed correct were positively related to volumes of right CA1 (r = 0.554, $p = 0.017$; r = 0.589, $p = 0.010$, respectively) and right CAt (r = 0.546, $p = 0.019$; r = 0.586, $p = 0.011$, respectively). PRM delayed correct was positively related to mean cortical thickness (r = 0.614, $p = 0.007$). SWM total error score was negatively related to total GM (r = -0.598, $p = 0.009$) and right SUB (r = -0.566, $p = 0.014$).

Functional Connectivity Motion Analysis

A second functional connectivity analysis, parallel to that described in the main document, was undertaken to assess the impact of individual motion estimates on our results. Datasets used in this analysis included an additional post-processing step. In addition to the post-processing described in the main document, individual volumetric residual motion estimates, output by SLOMOCO, were regressed from the resting state time series. The analysis then

proceeded as described in the main document. The resulting conjunction mask was essentially identical to that calculated for the main analysis, encompassing all of the same regions. As shown in Supplementary Table 3, the results of the group comparisons were also very similar. In the main analysis, connectivity from the left to right hippocampus was significantly weaker in the group with DS ($p < 0.0179$), but this difference did not survive FDR correction in the additional analysis ($p < 0.0417$). Otherwise, all of the same regional comparisons survived FDR correction, and all differences were similar in both magnitude and direction. Individual residual motion estimates do not appear to have a major impact on the results of our analysis.

Supplementary Table 1. Cognitive scores (mean ± standard deviation) for participants with DS in Protocol 2

	Analysis	
	Volumetric	Connectivity
n	18	10
CVLT Total	11.9 ± 6.5	11.1 ± 6.6
CVLT Recall	0.70 ± 0.76	0.69 ± 0.90
TROG	19.6 ± 5.5	18.7 ± 5.3
SSP	2.8 ± 1.7	3.1 ± 1.4
PRM Total	24.1 ± 6.4	23.6 ± 6.5
PRM Imm.	16.3 ± 4.2	16.4 ± 4.2
PRM Delay	7.7 ± 2.8	7.2 ± 3.0
SWM Error	66.3 ± 15.4	68.5 ± 18.8
SWM Strat.	38.3 ± 2.5	37.9 ± 2.5
PAL	7.6 ± 3.4	6.7 ± 3.4
DAS Digits	9.4 ± 4.0	8.7 ± 4.6
DAS Mat.	51.3 ± 10.5	48.8 ± 8.4
G/NG	519.9 ± 116.1	503.2 ± 125.5
PPVT	100.94 ± 26.6	98.6 ± 30.4

CVLT = California Verbal Learning Test; DAS = Differential Ability Scales; G/NG = Go/No-Go; PAL = Paired Associates Learning; PPVT = Peabody Picture Vocabulary Test; PRM = Pattern Recognition Memory; SSP = Spatial Span; SWM = Spatial Working Memory; TROG = Test for Reception of Grammar

Supplementary Table 2. Correlation coefficients between anatomical measures and cognitive function in 18 individuals with DS. Bold values survived correction for multiple comparisons ($p < 0.02$).

Region	CVLT Total	CVLT Recall	TROG	SSP	PRM Total	PRM Imm.	PRM Delay	SWM Error	SWM Strat.	PAL	DAS Digits	DAS Mat.	G/NG	PPVT
Whole Brain														
Cerebral WM	-0.060	-0.083	-0.347	0.285	-0.229	-0.206	-0.213	0.396	-0.104	-0.070	-0.263	-0.402	-0.479	-0.095
Lateral Ventricle	0.325	0.114	0.380	0.312	0.090	0.112	0.038	-0.370	0.154	0.038	0.405	0.201	-0.208	0.331
Total GM	-0.009	0.006	0.431	-0.001	0.196	0.174	0.187	**-0.598**	-0.164	-0.150	0.367	0.512	0.346	0.228
Subcortical GM	0.274	0.131	0.268	0.337	0.203	0.240	0.104	-0.160	-0.125	-0.046	0.227	0.245	-0.136	0.193
Cortical Thickness	-0.044	0.129	0.423	-0.140	0.534	0.402	**0.614**	-0.300	0.041	0.303	0.184	0.161	-0.071	0.157
Hippocampus, Left														
ERC	0.135	0.259	0.198	0.313	-0.057	-0.087	0.001	-0.415	-0.285	0.012	0.352	0.211	0.260	0.377
SUB	0.258	0.423	0.434	-0.041	0.398	0.405	0.301	-0.304	-0.391	0.203	0.401	0.503	0.206	0.346
CA1	0.054	0.246	0.110	-0.103	0.339	0.306	0.316	-0.014	-0.199	0.288	0.087	0.339	0.209	0.201
CAt	0.022	0.215	0.071	-0.079	0.292	0.255	0.283	-0.003	-0.220	0.279	0.062	0.307	0.190	0.181
DG	-0.065	0.149	-0.115	0.351	-0.110	-0.187	0.030	-0.099	-0.096	0.163	0.004	0.145	0.230	0.167
Tail	-0.028	0.113	0.026	0.238	-0.061	-0.115	0.032	-0.165	-0.511	0.065	0.201	0.082	-0.030	0.096
HIP	0.066	0.293	0.133	0.078	0.229	0.183	0.247	-0.139	-0.307	0.260	0.172	0.360	0.227	0.257
Hippocampus, Right														
ERC	0.140	0.082	0.201	0.422	-0.044	-0.028	-0.057	-0.131	-0.254	0.124	0.201	0.236	-0.390	0.399
SUB	0.083	0.135	0.360	0.246	0.118	0.041	0.194	**-0.566**	-0.216	-0.009	0.371	0.373	-0.012	0.193
CA1	0.107	0.081	0.023	-0.025	**0.554**	0.450	**0.589**	-0.343	0.386	0.477	0.006	0.339	0.166	0.115
CAt	0.098	0.076	0.029	-0.007	**0.546**	0.440	**0.586**	-0.356	0.370	0.475	0.014	0.336	0.164	0.120
DG	0.099	0.027	-0.194	0.145	-0.142	-0.099	-0.175	-0.255	0.032	0.073	0.033	0.074	0.098	0.209
Tail	-0.009	0.212	0.041	0.364	-0.147	-0.165	-0.089	-0.059	-0.494	-0.042	0.226	-0.263	-0.130	0.167
HIP	0.132	0.129	0.058	0.184	0.303	0.230	0.346	-0.533	0.117	0.322	0.174	0.340	0.127	0.251

CA1 = cornu Ammonis field 1; CAt = CA total; CVLT = California Verbal Learning Test; DAS = Differential Ability Scales; DG = dentate gyrus; ERC = entorhinal cortex; GM = grey matter; G/NG = Go/No-Go; HIP = total hippocampus; PAL = Paired Associates Learning; PPVT = Peabody Picture Vocabulary Test; PRM = Pattern Recognition Memory; SSP = Spatial Span; SUB = subiculum; SWM = Spatial Working Memory; TROG = Test for Reception of Grammar; WM = white matter

Supplementary Table 3. Group differences in residual motion-corrected connectivity to the left and right hippocampus (n = 22 in both groups).

*Results of Student's t-tests between groups. Values in bold survived the correction for multiple comparisons. **Regions where connectivity is stronger in DS as compared to controls.

			*p**	
	**Region**	**BA**	**Left**	**Right**
L	superior frontal gyrus	8	**1.7×10^{-5}**	**0.0003**
L	middle/superior frontal gyrus	8	0.1161	0.1191
R	middle/superior frontal gyrus	8	**0.0024**	**0.0051**
L	medial frontal gyrus	9	**0.0033**	**0.0016**
R	medial frontal gyrus	9	**0.0011**	**5.9×10^{-5}**
R	precentral gyrus	4	0.0706	**0.0051**
L	anterior cingulate	24	**0.0001**	**5.4×10^{-5}**
L	posterior cingulate	30	0.5628	0.7199
R	posterior cingulate	31	0.3907	0.3777
L	middle temporal gyrus	21	0.0361	0.1823
R	middle temporal gyrus	21	0.0325	**0.0050**
L	superior/middle temporal gyrus	39	0.5607	0.7285
R	superior/middle temporal gyrus	39	0.7550	0.3821
L	lingual gyrus	17	0.0288	0.0944
R	lingual gyrus	17	0.1259	0.0640
L	precuneus	7	0.1379	**0.0107****
L	hippocampus		-	**0.0074**
R	hippocampus		0.0417	-
L	parahippocampal gyrus	35	0.9863	0.5189
R	parahippocampal gyrus	35	0.8092	0.8676

BA = Brodmann area; L = left; R = right

Supplementary Figure 1. Mean hippocampal subfield volumes plotted by group (DS n = 34; HC n = 27). Error bars represent standard deviation and individual data points are included for each measure. See Table 1 for results of group comparisons.*p < 0.0010; **p < 0.0025. (CA1 = cornu Ammonis field 1; CAt = CA total; DG = dentate gyrus; DS = Down syndrome; ERC = entorhinal cortex; HC = healthy control; HIP = total hippocampus; ICV=intracranial volume; SUB = subiculum)

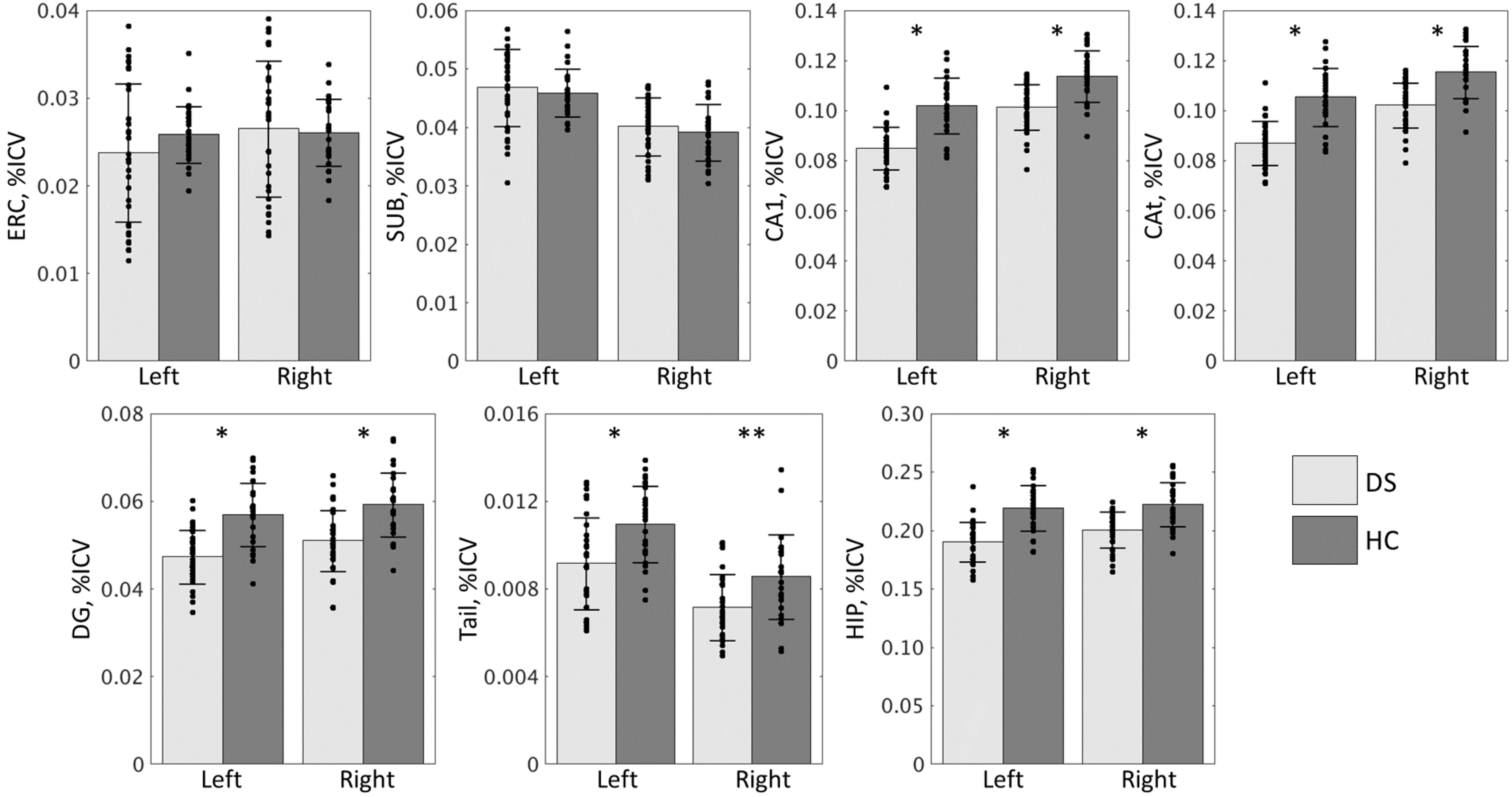

Supplementary Figure 2. The relationship of whole brain and hippocampal volumes to cognitive performance in individuals with DS (n = 18). All correlations were significant after FDR correction. (CA1 = cornu Ammonis field 1; CAt = CA total; GM = grey matter; ICV=intracranial volume; PRM=Pattern Recognition Memory; SUB = subiculum; SWM = Spatial Working Memory)

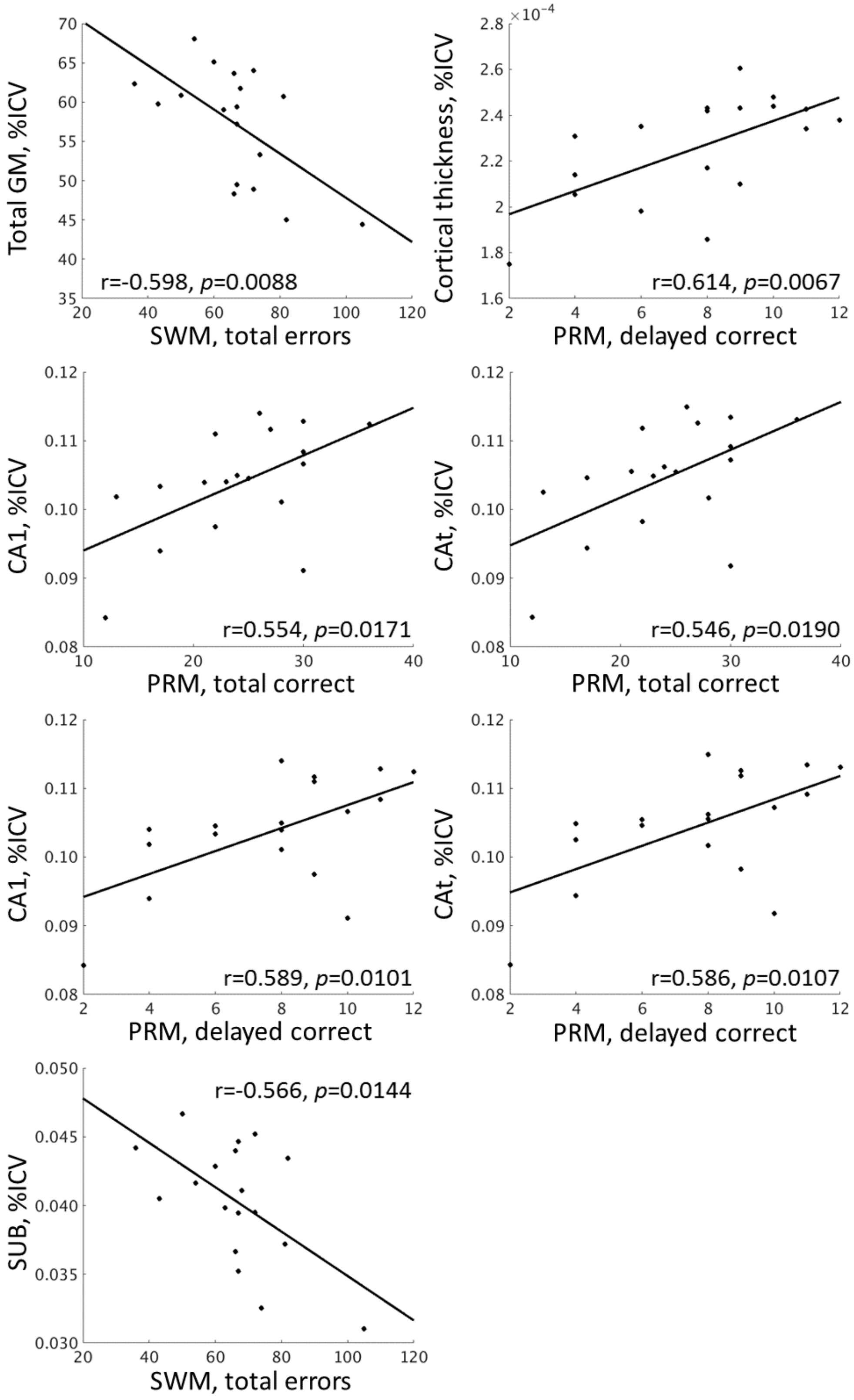

Supplement: fcab088_Supplementary_Data [file fcab088_supplementary_data.pdf]
